# Short-term safety and effectiveness of conversion from sleeve gastrectomy to Ring augmented Roux-en-Y gastric bypass

**DOI:** 10.1186/s12893-024-02552-7

**Published:** 2024-09-19

**Authors:** Kayleigh Ann Martina van Dam, Evelien de Witte, Pieter Petrus Henricus Luciën Broos, Jan Willem M. Greve, Evert-Jan Gijsbert Boerma

**Affiliations:** 1 Surgery, Zuyderland Medical Center, Henri Dunantstraat 5, Heerlen, 6419 PC The Netherlands; 2 Nederlandse Obesitas Kliniek (Dutch Obesity Clinic), Heerlen, The Netherlands; 3 Department of Surgery, Institute for Nutrition and Translational Research in Metabolism, NUTRIM, Maastricht University Medical Center, Maastricht, The Netherlands

**Keywords:** Sleeve gastrectomy, MiniMIZER, Ring augmented Roux-en-Y gastric bypass, Conversional surgery, Band, Banded RYGB, Ring

## Abstract

**Background:**

Weight recurrence, suboptimal clinical response and functional disorder (such as reflux) after a Sleeve Gastrectomy (SG) are problems that may require conversional surgery. For reflux, conversion to Roux-en-Y Gastric Bypass (RYGB) is considered effective. Regarding treatment for suboptimal clinical response, the technique of choice remains a subject of debate. This study aims to evaluate the safety and effectiveness of conversion from SG to Ring-augmented RYGB ( RaRYGB).

**Methods:**

All laparoscopic SG to RaRYGB conversions performed between January 2016 and January 2022 were included. Primary outcome was percentage total weight loss (%TWL) after 1-year follow-up. Secondary outcomes consisted of cumulative %TWL, complications (with a focus on ring-related complications), and resolution of medical-associated problems.

**Results:**

We included 50 patients of whom 44 were female. Mean pre-conversion BMI was 37.6 kg/m^2^. All patients have reached the 1-year follow-up point, however 10 were lost to follow-up. After 1-year mean TWL was 17.8% while mean cumulative TWL, calculated from primary SG, was 32%. A total of 10 complications occurred in 8 patients within 30 days, 6 of which were *≤* CD3a and 4 *≥* CD3b. One MiniMizer was removed for complaints of severe dysphagia. Of the 35 medical-associated problems present at screening 5 remained unchanged(14.2%), 15 improved(42.9%) and 15 achieved remission(42.9%).

**Conclusion:**

Our series of 50 patients undergoing conversion from SG to RaRYGB is adequate and successful regarding additional weight loss 1 year after conversion, cumulative weight loss, complication rate and achievement of improvement or remission of medical-associated problems.

**Supplementary Information:**

The online version contains supplementary material available at 10.1186/s12893-024-02552-7.

## Introduction

The most effective treatment in patients with obesity is bariatric surgery. Bariatric surgery results in long-term weight loss, remission of medical-associated problems and mortality reduction [1].

The most common performed procedure worldwide is the Sleeve Gastrectomy (SG) [2]. Although SG has been associated with favorable short- and medium-term weight loss outcomes, 20 to 30% of patients eventually require revisional or conversional surgery [3–5]. Indications for additional surgery after SG includes primary non-response, weight recurrence and gastrointestinal complaints (e.g., stenosis and/or severe gastro-oesophagal reflux disease (GERD)) [3]. The most performed surgical procedures after a SG are a re-sleeve or conversion to either RYGB or single-anastomosis duodeno-ileal bypass (SADI) [4]. A recent randomized controlled trial comparing the banded and non-banded re-sleeve gastrectomy showed a similar achievement of weight loss [5]. The banded re-sleeve showed a more stable weight loss after two years but was accompanied by more food intolerance. In addition, the re-sleeve is not as effective as other procedures, especially on the long-term. Conversion to RYGB has been shown to be especially effective regarding GERD [3, 4, 6–8]. However, previous research demonstrated a lower percentage total weight loss (%TWL) following conversion from SG to RYGB compared to primary RYGB [9]. In addition, weight recurrence can occur even after RYGB [10]. To further improve weight loss and minimize weight recurrence after RYGB a silicone ring can be added on the pouch above the gastrojejunal anastomosis [11, 12]. Multiple studies have shown the benefits of adding this silicone ring resulting in increased weight loss and less weight recurrence [11, 13–15]. One study demonstrated a significant 5% higher TWL up until 5 years after Ring augmented RYGB ( RaRYGB) compared to regular RYGB [11]. Nevertheless, the silicone ring can also cause complications of its own such as slippage, erosion and dysphagia [13]. To our knowledge there are no studies in current literature that describe the results of conversion from SG to RaRYGB.

The aim of the present study was to evaluate the short-term safety and effectiveness of conversion from SG to RaRYGB regarding weight loss, medical-associated problems, and complications.

## Methods

### Patient selection

All consecutive patients that underwent conversion from SG to RaRYGB between January 1st 2016 and January 31st 2022 in Hospital X were included in the present study. Indications for conversion were weight recurrence or complications such as stenosis or GERD categorized as gastrointestinal complaints. Current consensus on weight recurrence is defined as an increase of more than 30% of the initial weight loss or the return of an obesity-related medical-associated problem [16]. This definition is also maintained by the Dutch Obesity Clinic, however this was not yet the case during the conduction of the study. GERD was evaluated with the presence of complaints, PPI use and possibly an endoscopy. A stenosis was significant if it gave obstruction problems. The patients with suboptimal response were not categorized as a separate group as they all had accompanying functional problems. For all indications the RYGB is the preferred secondary procedure, and the MiniMizer is added by default for both primary and conversional RYGB procedures. All patients were pre-operatively screened and approved by a multidisciplinary team.

### Surgical procedure

All procedures were performed laparoscopically. Five trocars were placed and if necessary adhesiolysis was performed, especially between the gastric sleeve and the liver to ensure the placement of the liver retractor. First an 8–10 cm long pouch was created by transection of the sleeve and was resized over a 40 French orogastric tube. If a hiatal hernia was present, cruroplasty was performed. The jejunum was identified at the ligament of Treitz and the biliopancreatic limb was measured at a length of 60 cm in all patients. The limb was brought antecolically and antegastrically to the gastric pouch and a linear stapled end-to-side gastrojejunal anastomosis was created. The biliopancreatic limb was transected and a side-to-side jejunojejunal anastomosis was created with an alimentary limb of 120 cm. Both mesenteric defects were closed using endoclips. A silicone ring, the MiniMizer (Bariatric Solutions International, Switzerland), was placed around the pouch. The MiniMizer was placed at least 2 cm above the gastrojejunal anastomosis and at least 2 cm below the gastroesophageal junction. The closing position was standardized at 7.5 cm for males and 7.0 cm for females. The MiniMizer was fixated on the vertical staple line with a non-absorbable suture. Post-operatively patients follow an obligated five-year postoperative trajectory at the Dutch Obesity Clinic.

### Data collection

All data were retrospectively collected from electronic patient files at Hospital X. The baseline data included age, gender, height, weight, BMI, medical-associated problems, and conversion indications at screening. The medical-associated problems comprised hypertension (HT), diabetes mellitus (DM), obstructive sleep apnea syndrome (OSAS), GERD, and dyslipidemia defined according to the standardized outcomes in bariatric surgery [17]. The presence of a medical-associated problem was evaluated during screening by following the definitions of the ASMBS Outcome Reporting Standards [17]. For all medical-associated problems the symptoms and/or use of medication were evaluated to determine if a problem was present. Peri-operative data of the conversional procedure included surgery duration (min), simultaneous hiatal hernia repair, and closing position of MiniMizer. A hiatal hernia was identified during the surgery and treated by cruroplasty if any part of the stomach extended through the diaphragmatic opening.

The primary outcome measure of %TWL after 1 year follow-up was calculated using the weight after 1 year of FU compared to the weight prior to the conversional procedure. The secondary outcomes consisted of cumulative %TWL, early (< 30 days) and late (> 30 days and *≥* 1 year) complications and included both general and ring related complications, and resolution of medical-associated problems. The cumulative %TWL was calculated using the initial weight during screening for the SG procedure. Classification of the complications was performed according to the Clavien-Dindo classification [18]. The resolution of a medical-associated problem was categorized as remission, improved, unchanged, worsened or not applicable. Specifically for GERD the resolution was defined as follow:

- Remission: absence of symptoms, no medication use, and normal results on physiological tests (e.g., 24–48 h pH monitoring or endoscopy).
- Improved (objective): reduction in symptoms, decreased medication use, and/or improvement on physiological tests (24–48 h pH monitoring or gastro-duodenoscopy).
- Improved (subjective): reduction in symptoms and/or decreased medication use.
- Unchanged: no remission or improvement as previously described.
- Worsened: Worsening of symptoms and/or initiation or resumption of medication after a period of absence.

### Hypotheses

It is expected that the conversion from SG to RaRYGB results in a %TWL after one-year that is superior to the weight loss achieved with primary standard RYGB. In addition, the conversion is hypothesized to lead to a complication rate comparable to the primary standard RYGB and other conversional procedures after SG. The ring-related complications such as dysphagia, slippage and erosion are expected to be low.

### Statistical analysis

Statistical analysis was performed using the IBM SPSS Statistics for Windows, version 26.0. Categorical variables were presented as frequencies with percentages. Continuous variables were presented as mean ± standard deviation (SD) for normal distributed variables and median and inter-quartile-range (IQR) for a skewed distribution. Differences between subgroups were tested using a Student’s *t-test* or a Mann-Whitney-U test. A *p*-value of *p* < 0.05 was considered statistically significant. Missing data were reported as such.

For this retrospective data study, local approval was given by the local ethics committee in accordance with the ethical standards as stated in the 2013 Declaration of Helsinki.

## Results

A total of 50 patients were analyzed of whom 44 were female (88%) and 6 male (12%). The preoperative demographical data at screening for conversion are summarized in Table 1. The group had a mean age of 44 years (± 10.3), and median preoperative BMI was 37.6 kg/m^2^ (33.4–40.8). The medical-associated problems consisted of HT (18%), DM (2%), OSAS (8%), GERD (36%) and dyslipidemia (6%).

The indications for conversion were weight recurrence (40%) or gastrointestinal complaints (60%). Of the patients with gastrointestinal complaints the majority also experienced recurrent weight gain (60%). The median operating time for the conversional surgery was 69 min (57–97). In 22 (44%) of the patients a hiatal hernia (HH) repair was performed simultaneously with the RaRYGB conversion. In the conversion group with gastro-intestinal problems 50% had simultaneous HH repair while 35% of the weight recurrence group also underwent a simultaneous HH repair. The closing position of the silicone MiniMizer ring varied between 7 and 7.5 cm diameter. Twenty-nine patients had a closing position of 7 cm and 21 of 7.5 cm. Of the male patients 83.3% had a MiniMizer closing position of 7.5 cm while 63.6% of the females had a closing position of 7 cm.

**Table 1:** Baseline characteristics

Baseline characteristics	*N* = 50
Age (years)	44 *±* 10.3
Gender	
*Male*	6 (12)
*Female*	44 (88)
Height (cm)	168.1 *±* 6.5
Weight at screening (kg)	107 *±* 24.4
BMI at screening (kg/m^2^)	37.7 *±* 7.6
Medical-associated problems at screening	
*Hypertension* *Diabetes mellitus* *OSAS* *GERD* *Dyslipidemia*	9 (18) 1 (2) 4 (8) 18 (36) 3 (6)
Surgery duration	69 (57–97)

Data are presented as mean ± standard deviation, median (IQR) or N (%)

### Effect on weight

Mean BMI at screening for SG was 45.9 kg/m^2^ (*±* 8.4) while the mean BMI at screening for the conversion was 37.7 kg/m^2^(+ *7.6)*. The 1-year follow-up point was reached by all patients while data was available for 40/50 (80%) of patients. Of the 40 patients, the mean %TWL after 1-year follow-up calculated from the conversion was 17.8 *±* 10 as shown in Table 2. The weight loss resulted in a mean BMI of 31.1 kg/m^2^ *±* 6.7 after 1-year.

Table 2 also presents the cumulative TWL from the initial surgery. At the moment of screening for conversion, patients had a mean %TWL of 17.9 (*±* 13.4). After the 1-year follow-up the cumulative %TWL, calculated from the primary surgery was 32 *±* 12.9.

**Table 2:** %TWL and cumulative %TWL during 1-year follow-up after conversion

	Follow-up	BMI (kg/m^2^)	%TWL from revision	(cumulative) %TWL from primary surgery
Primary surgery (SG)	50 (100)	45.9 *±* 8.4	-	-
Pre-conversion	50 (100)	37.3 *±* 7.2	-	17.9 ± 13.4
3 month follow-up	46 (92)	33.5 ± 6.3	11.3 ± 4.6	26.5 ± 12.2
6 month follow-up	40 (80)	31.8 ± 6.4	14.5 ± 6.9	30.3 ± 12.4
12 month follow-up	40 (80)	31.1 ± 6.7	17.8 ± 10	32 ± 12.9

**Fig. 1:**
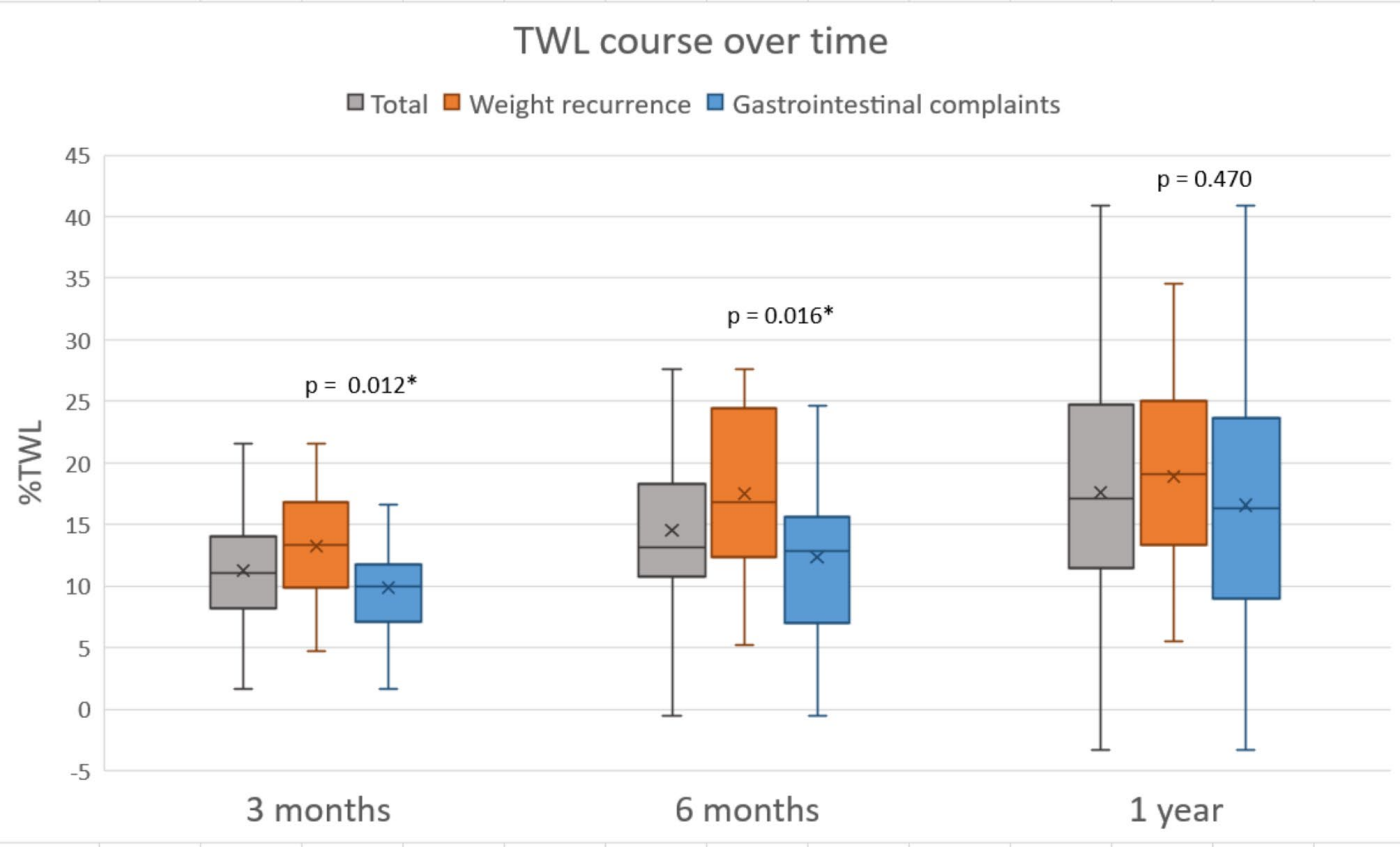
%TWL over 1-year follow-up. *P*-value calculated with the Student’s t-test

The effect on weight was also compared for the subgroups, based on the indication for conversion as shown in Fig. 1. The patients who were operated due to weight recurrence had a significant higher mean BMI of 40.7 *+ 7.2* kg/m^2^ before conversion compared to a BMI of 36.2 *±* 7.2 kg/m^2^ for the patients with gastrointestinal complaints, *p* = 0.017. At 1-year follow-up the %TWL was 18.9 *±* 8.2 for the weight recurrence group and 16.6 *±* 11.2 for the gastrointestinal complaints group, *p* = 0.470.

### Complications

A total of 17 patients with short- and long-term complications were registered (Table S1, additional file). In these 17 patients there were a total of 22 complications, of which 10 occurred in 8 patients (16%) within 30 days. Of the short-term complications 6 were classified as Clavien-Dindo (CD) of *≤* CD3a and 4 were classified as *≥* CD3b as shown in Table 3. The short-term complications consisted of internal herniation, anastomotic stenosis, anastomotic leakage, anastomotic bleeding, wound infection, and intra-abdominal abscess formation. Of these, 6 complications required reinterventions namely 4 laparoscopically (CD3b) and 2 endoscopically (CD3a). In all but one of the complications either prolonged admission or readmission was required.

Within the first year three patients had a MiniMizer related complication (6%). Two patients had slippage of the ring which was corrected surgically by repositioning of the ring. One patient had dysphagia complaints without signs of erosion or slippage of the ring resulting in the MiniMizer being laparoscopically removed. At 1-year follow-up the MiniMizer was still in situ in 47 of the patients. In addition to the removal due to dysphagia the MiniMizer was removed within the 30-day post-operative time frame as part of the surgical treatment of anastomotic leakage in two patients.

**Table 3:** Short-term complications

Variables	Conversion (*N* = 50)
MiniMizer in situ	47 (94)
MiniMizer related complications	
*Ringslippage* *Ring erosion* *Small bowel obstruction* *Other (dysphagia)*	2 (4) 0 0 1 (2)
Patients with short-term complications	8 (16)
Short-term (*≤* 30 days) complications according to Clavien Dindo	
*1* *2* *3a* *3b*	1 (2) 3 (6) 2 (4) 4 (8)
Short-term complication related hospital admission	
*No admission* *Prolonged admission* *Readmission*	1 (2) 2 (4) 7 (14)

### Obesity medical-associated problems

During screening for conversion, the prevalence of the associated medical problems was available in all 50 patients and resulted in 35 medical problems being present. For HT the prevalence was 18%, for DM 2%, for OSAS 8%, for GERD 36% and for dyslipidemia 6%. Of the 35 medical problems at 1-year follow-up 5 (14.2%) remained unchanged, 15 (42.9%) improved and 15 (42.9%) achieved remission (Table 4).

**Table 4:** Medical-associated problems at screening for conversion and 1-year follow-up

		Evolution after 1 year		
	Medical-associated problems prevalence	Unchanged	Improved	Remission
Hypertension	9 (18)	3 (33.3)	1 (11.1)	5 (55.6)
Diabetes	1 (2)	-	-	1 (100)
OSAS	4 (8)	-	3 (75)	1 (25)
GERD	18 (36)	1 (5.6)	10 (55.6)	7 (38.8)
Dyslipidemia	3 (6)	1 (33.3)	1 (33.3)	1 (33.3)

## Discussion

Studies regarding the conversion from SG to RYGB describe a %TWL ranging from 10.1 to 22.8% after at least 1 year follow-up [4, 7, 19]. The overall %TWL after 1 year of this study was 17.8% which is in accordance with the previous mentioned range. In addition, %TWL found in the present study is comparable to other conversional procedures, namely from SG to BPD/DS (14%) and to SADI (21.5%) [4, 20]. However, comparison is difficult as the results are partly dependent on the remaining weight loss after the primary procedure. Therefore, one should consider cumulative %TWL rather than %TWL after conversion. However, the cumulative %TWL is usually not reported in literature. Of the before mentioned papers only D’Urso et al. reported a cumulative %TWL of 29.3 [19]. The present study shows a cumulative %TWL of 32, which is comparable to the %TWL of 30 in 2420 patients with a primary standard RYGB [21].

For the subgroups based on the conversion indication of either weight recurrence or gastrointestinal complaints there was no significant difference between achieved weight loss at 1-year follow-up (%TWL 18.9 vs. 16.6). The subgroup with weight recurrence has a higher %TWL of 18.9 compared to the study of Landreneau et al. who demonstrated a %TWL of 16.1 in patients who were converted due to weight recurrence [22]. In comparison to the %TWL of 19.3% after 3 years in the study of Quezada et al. the present study has similar results [23].

This study shows a short-term complication rate of 16% within the first 30-days after surgery. Of the short-term complications 40% were minor (CD < 2) and 60% was severe (*CD > 3a). The* MiniMizer related complications were low with three patients (6%) who had either slippage of the ring or dysphagia complaints. The overall complication rate in the present study is comparable to primary RYGB and lower than RYGB as a conversional procedure both in total and per severity. For the primary RYGB procedure the complication rate varies between 6.3 and 6.5% with one outlier study having a high complication rate of 27.4% [24–26]. As a conversional procedure, the conversion from SG to standard RYGB has a complication rate varying between 22 and 31.5% (22, 27 ). Furthermore, the present study shows a complication rate equal or lower compared to conversion to DS (14.2%) and SADI (42%) based on the systematic review by Franken et al. [27]. Only the re-sleeve gastrectomy and the one-anastomosis gastric bypass (OAGB) had lower complication rates of 6.7 and 6% [27]. Possible reasons for the lower complication rates in the previous two techniques is attributed to the fact that only major complications (*≥* CD3) are included in the previously mentioned percentages. Other reasons could be a shorter operating time, a simpler surgical technique, and fewer anastomoses.

Regarding the medical-associated problems, the current study showed that in 85.8% of the problems improvement or remission occurred. A systematic review comparing resolution of the medical-associated problems between RYGB and SG showed a significant higher resolution regarding HT, dyslipidemia, and GERD after RYGB [28]. The current study includes both improvement and resolution, and these combined are comparable to the results of the study of Yorke et al. which has an overall resolution rate of 88.8% after conversion from SG to standard RYGB [29]. It should be noted that the study of Yorke only focused on total resolution defined as no more usage of medication, while this study distinguished between improvement (e.g., cessation of one medication instead of all medications) and resolution.

The findings of our study suggest that conversion from SG to RaRYGB is safe and effective with favorable short-term outcomes. The RaRYGB seems especially suitable for patients experiencing weight recurrence. The systematic review and meta-analysis of Pavone et al. compared standard and RaRYGB and showed a significant increase in percent excess weight loss (%EWL) [30]. In addition, the postoperative complications were not significantly increased. However, it should be noted that the included studies use several types of bands/rings among which the MiniMizer but also polypropylene mesh. This is in accordance with the results of this study where the ring-related complications are low. Regarding GERD, a systematic review indicates that RYGB is more effective than SG in resolving GERD symptoms [28]. Our study aligns with these with an improvement of GERD in 94.4% and shows the RaRYGB is non-inferior to the standard RYGB. Therefore, the MiniMizer can be beneficial for many patients.

### Limitations

First, the retrospective setting should be considered when interpreting the results. The follow-up rate after bariatric surgery is often a problem. In the present study a one-year follow-up rate of 80% was achieved, which is comparable to the literature as the rate of one year follow-up varies between 63 and 98.3% (21, 25 ). The loss to follow-up is attributable to patients that were no longer following the (obligated) postoperative trajectory. In addition, some patients were operated in our hospital only for the conversional surgery while the primary procedure was performed elsewhere in the country or even abroad. Part of the loss to follow-up can be attributed to patients returning to their own region.

Other limitations are the small sample size and the relative short follow-up period. Although we believe that the 1-year follow-up is sufficient for initial outcomes and (short-term) complications, a longer follow-up period would be interesting as a follow-up study to assess long term weight loss results and late adverse events (especially ring-related complications). Moreover, the absence of standard endoscopic evaluations to objectively assess GERD complaints should be considered a limitation. The GERD symptoms are considered an indication for conversion surgery.

## Conclusion

This is the first study that demonstrates the safety and effectiveness of a conversion from SG to a RaRYGB. The complication rates were low, and the significant cumulative weight loss achieved at 1-year follow-up was comparable to primary RYGB while resolution of medical-associated problems was comparable to conversion from SG to standard RYGB.

## Electronic supplementary material

Below is the link to the electronic supplementary material.

Supplementary Material 1

## Data Availability

Data is provided within the manuscript.
