# Supplementary material for: Short-term safety and effectiveness of conversion from sleeve gastrectomy to Ring augmented Roux-en-Y gastric bypass
Source: BMC Surg. 2024 Sep 19;24:266. doi: 10.1186/s12893-024-02552-7 (PMC11411827; doi:10.1186/s12893-024-02552-7)
Supplement: Supplementary file 1 — Supplementary Material 1 [file 12893_2024_2552_MOESM1_ESM.docx]

Additional file 1.

***Table S1. Short- and long-term complications***

| Variables | Conversion  (N = 50) |
| --- | --- |
| MiniMizer in situ | 47 (94) |
| MiniMizer related complications  *Band slippage*  *Band erosion*  *Small bowel obstruction*  *Other (dysphagia)* | 2 (4)  0  0  1 (2) |
| Patients with complications (total)  Patients with short-term complications | 17 (34)  8 (16) |
| Short-term (< 30 days) complications according to Clavien Dindo  *1*  *2*  *3a*  *3b* | 1 (2)  3 (6)  2 (4)  4 (8) |
| Long-term (>30 days) complications according to Clavien Dindo  *2*  *3a*  *3b* | 1 (2)  2 (4)  9 (18) |
